# An all phosphorene lattice nanometric spin valve

**DOI:** 10.1038/s41598-024-58589-4

**Published:** 2024-04-21

**Authors:** P. Kumari, S. Majumder, S. Kar, S. Rani, A. K. Nair, K. Kumari, M. Venkata Kamalakar, S. J. Ray

**Affiliations:** 1 Department of Physics, Indian Institute of Technology Patna, Bihta, 801103 India; 2 Department of Physics and Astronomy, Uppsala University, Box 516, 75120 Uppsala, Sweden

**Keywords:** Nanoelectronics, Spintronics, 2D magnet, Spintronics, Two-dimensional materials, Electronic and spintronic devices

## Abstract

Phosphorene is a unique semiconducting two-dimensional platform for enabling spintronic devices integrated with phosphorene nanoelectronics. Here, we have designed an all phosphorene lattice lateral spin valve device, conceived via patterned magnetic substituted atoms of 3d-block elements at both ends of a phosphorene nanoribbon acting as ferromagnetic electrodes in the spin valve. Through First-principles based calculations, we have extensively studied the spin-dependent transport characteristics of the new spin valve structures. Systematic exploration of the magnetoresistance (MR) of the spin valve for various substitutional atoms and bias voltage resulted in a phase diagram offering a colossal MR for V and Cr-substitutional atoms. Such MR can be directly attributed to their specific electronic structure, which can be further tuned by a gate voltage, for electric field controlled spin valves. The spin-dependent transport characteristics here reveal new features such as negative conductance oscillation and switching of the sign of MR due to change in the majority spin carrier type. Our study creates possibilities for the design of nanometric spin valves, which could enable integration of memory and logic elements for all phosphorene 2D processors.

## Introduction

Black Phosphorous (BP), an allotropic form of phosphorus, is a layered material. Its relevance as a two-dimensional semiconductor that for high efficiency field effect transistors first emerged in experimental studies in 2014^1,2^. Since then, BP and its single layer form phosphorene have attracted tremendous theoretical and experimental interest. In comparison to graphene and dichalcogenide based 2D semiconductors, phosphorene exhibits both bandgap as well as a high carrier mobility^1,3^, which is quite unique. The bandgap in phosphorene is tunable by the number of layers as well as ambipolar transport can also be realized in BP^4,5^. Furthermore, its electrical properties are highly anisotropic^2^. All these special characteristics not only demonstrate phosphorene as an ideal candidate for nanoelectronics, but also for optoelectronic applications due to it’s direct bandgap. From the view point of spintronics, graphene always remained a superior candidate for spin transport due to its low spin-orbit coupling and high mobility^6,7^. On the other hand, the relatively low atomic mass of phosphorous atoms in phosphorene lattice implies a considerably low-spin orbit coupling, which makes it conducive for spin polarized electron transport. Realizing phosphorene based spintronics could enable the integration of memory and logic components for power efficient computing. Initial studies conducted in this direction showed spin transport feasibility in phosphorene^9,10^, direct demonstration of gate tunable spin transport^3^. Overall, today, the feasibility of phosphorene as a spin current carrying medium is established^8^. Further to it’s spin carrying capability, phosphorene also exhibits the prospects of being transmuted into a magnetic 2D material, which makes the feasibility of designing all phosphorene spintronics exceptionally exciting. To uncover such possibility, in this work, we perform an effort of making an all phosphorene spin device by designing patterned substituted in phosphorene (Ph) using several 3d-block atoms and calculate the spin filtering capabilities.

Here, we optimize magnetic properties of phosphorene by replacing the phosphorous atom with different atomic concentrations of a series of 3*d*-block metals. Our First-principles investigation show striking bi-polar magnetic semiconducting and half-metallic behaviour, which can be extensively tuned by a transverse electric field. Using such magnetic phosphorene (mPh), we make calculations to uncover the behaviour of mPh-Ph-mPh spin device, studying influence of varying kinds of magnetic impurities on spin polarised transport and reveal high efficiency magnetoresistance. Further control over the magneto-transport behaviour can be achieved by electric field control via introducing a local gate voltage. Extensive calculations reveal a phase diagram that show the variation of MR with bias voltage and substituted atom type is obtained, relating it with the electronic nature of the substituted system. Our results offer a new phase engineered design for building novel 2D spin circuits of high significance for all phosphorene computing.

**Figure 1:**
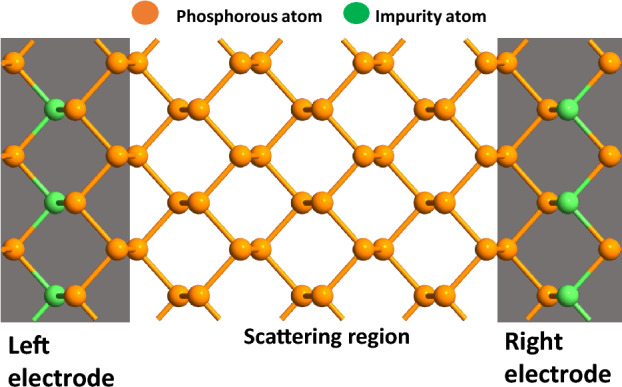
A schematic representation of a transition metal (impurity) atom doped phosphorene spin valve.

## Computational details

First-principles based investigation of the electronic properties of substituted Phosphorene was performed through density functional theory (DFT) calculations using Atomistic Toolkit^11^. The Spin-polarised self-consistent calculations were performed under the generalised gradient approximation (GGA) of the Perdew-Burke-Ernzerhof (PBE) exchange-correlation functional^12^. The double$-\zeta$ polarised basis-set was used for the expansion of electronic wave-function under the periodic boundary conditions. A Monkhorst–Pack *k*-grid^13^ of 9 × 9 × 1 was used for sampling the Brillouin Zone with an energy cut-off limit of 180 Ry. In order to reduce the interactions between neighbouring layers, a minimum vacuum space of 15 Å  was used in the non-periodic direction within the supercell. All the structures considered in the substituted/pristine configuration were relaxed until the atomic force on each atom is lesser than 10^-3^ eV/Å  in the equilibrium condition.

The transport properties in the 2-probe geometry was investigated using the Non-equilibrium Green’s Function (NEGF) formalism combined with DFT methodology^14,15^. In this process, the Hamiltonian of the 2-probe configuration is constructed in the presence of a finite voltage *V* between the semi-infinite left and right electrodes. The retarded Green’s function matrix $G_{\sigma }(\epsilon )$ using the hamiltonian can be expressed using Eq. (1),

1 $$\begin{aligned} G_{\sigma }(\epsilon ) = \frac{1}{(\epsilon + i\delta )S_{\sigma } - \mathcal {H_{\sigma }} - \Sigma _{L,\sigma }(\epsilon ) - \Sigma _{R,\sigma }(\epsilon )} \end{aligned}$$

where $\sigma , \epsilon , S_{\sigma }, \mathcal {H_{\sigma }}$ represent spin, energy, overlap and hamiltonian matrices respectively. The self-energy matrices for the left and right electrodes are represented by $\Sigma _{L,\sigma }(\epsilon ), \Sigma _{R,\sigma }(\epsilon )$ respectively. The density matrix ($D_{\sigma }$) and its components ($D_{\sigma }^L, D_{\sigma }^R$) can be estimated using $G_{\sigma }(\epsilon )$ by Eqs. (2–3),

2 $$\begin{aligned} D_{\sigma }^L = \frac{1}{2\pi }\int G_{\sigma }(\epsilon )f(\epsilon - \mu _L)\Gamma _{\sigma }^L(\epsilon )G_{\sigma }^{\dagger }(\epsilon )d\epsilon \end{aligned}$$

3 $$\begin{aligned} D_{\sigma }^R = \frac{1}{2\pi }\int G_{\sigma }(\epsilon )f(\epsilon - \mu _R)\Gamma _{\sigma }^R(\epsilon )G_{\sigma }^{\dagger }(\epsilon )d\epsilon \end{aligned}$$

$f{(\epsilon )}$ is the Fermi function, $\mu _L, \mu _R$ are the chemical potential of left (*L*) and right (*R*) electrodes respectively, with $\mu _L - \mu _R = eV$. The broadening of the electrodes are given by Eqs. (4–5),

4 $$\begin{aligned} \Gamma _{\sigma }^{L}(\epsilon ) = i[\Sigma _{L,\sigma }(\epsilon ) - \Sigma _{L,\sigma }^{\dagger }(\epsilon )] \end{aligned}$$

5 $$\begin{aligned} \Gamma _{\sigma }^{R}(\epsilon ) = i[\Sigma _{R,\sigma }(\epsilon ) - \Sigma _{R,\sigma }^{\dagger }(\epsilon )] \end{aligned}$$

The electron density $n_{\sigma }({\textbf {r}})$ in the basis function $\phi _{\nu }({\textbf {r}})$ can be calculated by Eq. (6),

6 $$\begin{aligned} n_{\sigma }({\textbf {r}}) = \sum _{\mu ,\nu }\phi _{\mu }({\textbf {r}})Re[(D_{\sigma })_{\mu ,\nu }]\phi _{\nu }({\textbf {r}}) \end{aligned}$$

For the self-consistent calculations, the process was repeated over iteratively for reaching convergence in $n_{\sigma }({\textbf {r}})$. The current was estimated using the Landauer–Büttiker formula^16,17^ as given in Eq. (7),

7 $$\begin{aligned} I_{\sigma } = \frac{2e}{\hbar }\int _{-eV/2}^{eV/2}T_{\sigma }(\epsilon ,V)[f_L(\epsilon -\mu _L) - f_R(\epsilon -\mu _R)]d\epsilon \end{aligned}$$

Here $T_{\sigma }(\epsilon )$ represents the transmission function that can be expressed in terms of $G_\sigma (\epsilon )$ and $\Gamma _{\sigma }$ by Eq. (8),

8 $$\begin{aligned} T_{\sigma }(\epsilon )= Tr [\Gamma _{\sigma }^R(\epsilon )G_{\sigma }^{\dagger }(\epsilon )\Gamma _{\sigma }^L(\epsilon )G_{\sigma }(\epsilon )] \end{aligned}$$

The *k*-point grid used for the transport calculation is 9 × 9 × 300 with 300 along the transport direction as no changes were observed beyond this limit.

## System description

The central structure of Phosphorene employed here consists of a $3 \times 4$ supercell of the same as used here^18^. In common spin valve devices, the interface oxide layers like MgO, Al_2_O_3_ are used as tunnel barrier with Co, Ni etc. as FM electrodes. Due to the presence of different materials, the nature of the interface is very crucial to minimise the strain and the abrupt change in material conductivity, that are key hurdles in achieving a high MR and to achieve a high MR ratio. The proposed all phosphorene planar spin valve structure intrinsically bypasses such challenge. The entire structure is made on a single phosphorene block with the two sides substituted heavily with 3d block elements, to make them ferromagnetic (FM) in nature which does not require any additional consideration of strain originating at the interfaces. The stability of the substituted structure in various substitutional configurations was confirmed by estimating the formation energy as shown in Sect. S2 of supporting information (SI)^22^ and the relative stability is also discussed with respect to other compounds that can be formed with the same set of atoms in Sect. S5^22^. A comparison with graphene in similar substitutional configurations revealed a lower formation energy for the case of phosphorene in most of the cases, which is illustrated in Fig. S6 in the SI^22^. The absence of imaginary bands in sample phonon band structures (refer to Fig. S7^22^) demonstrates dynamic stability for substitutional configurations. A sample schematic of the lateral spin valve structure used for this work is shown in Fig. 1, where the electrodes are heavily transition metal atoms substituted and the central scattering region is unsubstituted phosphorene. Comprehensive investigation was performed using a sub-total of ten different substitutional elements namely Sc, Ti, V, Cr, Mn, Fe, Co, Ni, Cu, and Zn in increasing order of atomic number (Z). Experimentally such doped structures can be fabricated via scanning tunneling microscopy (STM) based lithography technique as similar precise engineering was used in the fabrication of quantum dots on a P-doped Si wafer^37^ and controlled positioning of CO molecules on a Cu (111) surface^20^ in an ultrahigh vacuum environment.

In order to calculate the magnetoresistance (MR), the parallel (P) and anti-parallel (AP) configuration of the spin alignment of the FM regions in the structure were considered. The MR is estimated using the ’optimistic’ and ’pessimistic’ definitions given by,

9 $$\begin{aligned} \mathrm {MR_O = \frac{I_{P}- I_{AP}}{I_{AP}} \times 100\%} \end{aligned}$$

10 $$\begin{aligned} \mathrm {MR_P = \frac{I_{P}- I_{AP}}{I_{P}+I_{AP}} \times 100\%} \end{aligned}$$

where $\mathrm {I_{P}/I_{AP}}$ are the currents in the P/AP configurations of the FM electrodes respectively. The optimistic definition allows the MR value to vary from $- \infty$ to $+ \infty$, while the range is normalised between − 100% to + 100% in the pessimistic definition.

**Figure 2:**
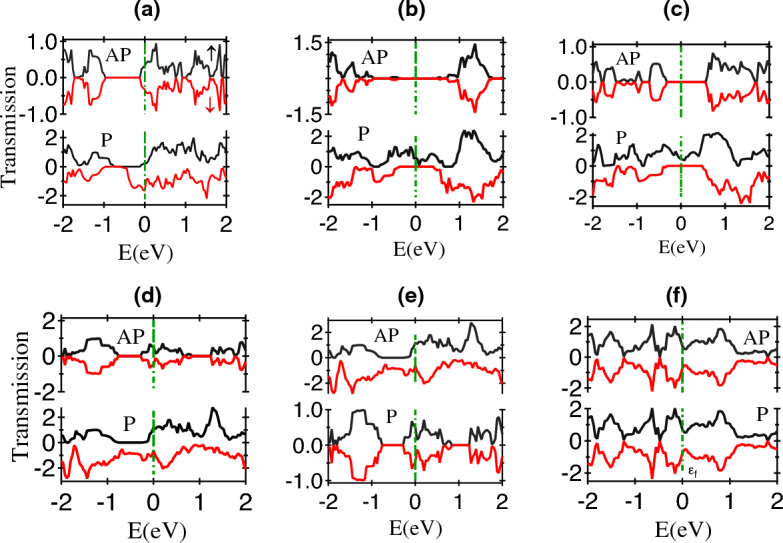
The Transmission spectrum at the zero bias voltage in P/AP configuration for various substitutional configurations of phosphorene spin valve: (**a**) Ti-Phosphorene (**b**) V-Phosphorene (**c**) Cr-Phosphorene (**d**) Mn-Phosphorene (**e**) Fe-Phosphorene (**f**) Co-Phosphorene.

## Results and discussions

This section is organised as follows: first we discuss the transport behaviour and MR response of the MTJ structure at zero-bias and at a finite-bias. Next, we explain the connection between the electronic properties of the electrodes and central scattering region with the observed MR signal. Finally, the role of a gate voltage is discussed for tuning the MR response. Details of the critical temperature of the electrodes can be found in Sect. S5 in the SI^22^, where the spin direction stays out of plane.

### Zero-bias results

The calculated MR values estimated for various substitutional configurations in the zero-bias configuration are given in Table 1. For Sc, Ni, Cu, and Zn, no MR is observed as there is no differences in transmission between then P and AP configuration. A very large value of MR is estimated in the presence of V and Cr-substituted cases with a value $\sim 10^6$ in the optimistic definition. It corresponds to a pessimistic MR $\sim$ 99.9% in both these cases, which is almost close to the perfect spin filtering performance. The MR values are also estimated by changing the substitution position and slightly increasing the substitution concentration of V—atoms in the electrode region, which are listed in Tables S4, S5 in SI. It was observed that the MR behaviour stays unaffected by such changes, demonstrating the robustness of the MR response. The origin of the high MR is related to the selective spin transmission as revealed by the spin resolved DOS shown in Fig. 2. In the P configuration, there is finite transmission around the Fermi level ($E_F$) for both the spin channels, while in the AP configuration both the transmission components are almost absent from both the spin channels which is same in the case of V and Cr as shown in Fig. 2(b, c). Significantly high values of MR are estimated in the presence of Ti, Mn, Co and Fe substitutional atom, indicating the effect of unpaired 3d electrons contributing to an enhanced spin polarization from the electrodes. The transmission spectrum in these cases in Fig. 2 shows finite transmission in both the P/AP cases around $E_F$, with the differences contributing towards the observed MR values.

The high MR values hold promise for a broad spectrum of applications, particularly in MR sensors. One approach to translate our findings into practical applications involves the development of lateral heterostructures using growth techniques. Such heterostructures can be fabricated through methods like chemical vapor deposition. Previous studies have successfully achieved similar heterostructures in materials like graphene and hBN^25^. Additionally, selective doping of black phosphorus presents another pathway. Techniques such as sputtering can be employed for this purpose^19^. The nanopatterning of black phosphorus can be achieved using block polymers, enabling the creation of various stripe patterns^32^. In these, extremely narrow regions can be selectively exposed and doped. The juxtaposition of pristine and doped black phosphorus stripes could lead to a novel amalgamation of multiple spin valves. We anticipate that these methods will not only facilitate the realization of our results on a larger scale but also are likely to result in unique magnetoresistance characteristics and values. They can further be interfaced with 2D nanomagnets to acheive room temperature spin-transport operation through the application of strain^33,35^ and twist engineering^30^.

**Figure 3:**
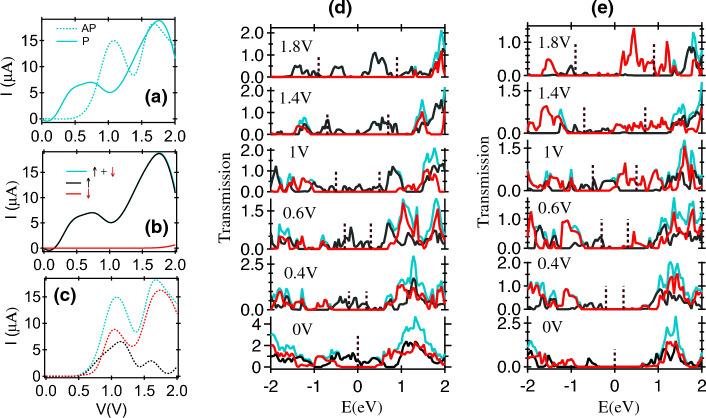
The I–V characteristics of V-based MTJ structure; (**a**) Total current in P/AP configuration (**b**) spin-polarized current in P configuration (**c**) spin-polarized current in AP configuration. Transmission spectrum at various applied voltages in the (**d**) P configuration (**e**) AP configuration.

**Table 1:** The MR ratio at zero bias voltage for various substitutional atoms.

Substitutional element	MR (optimistic) (%)	MR (pessimistic) (%)
Sc	0	0
Ti	250.03	55.56
V	2307807.74	99.99
Cr	1325589.14	99.98
Mn	444.59	68.67
Fe	123.89	38.25
Co	496.39	71.28
Ni	0	0
Cu	0	0
Zn	0	0

**Figure 4:**
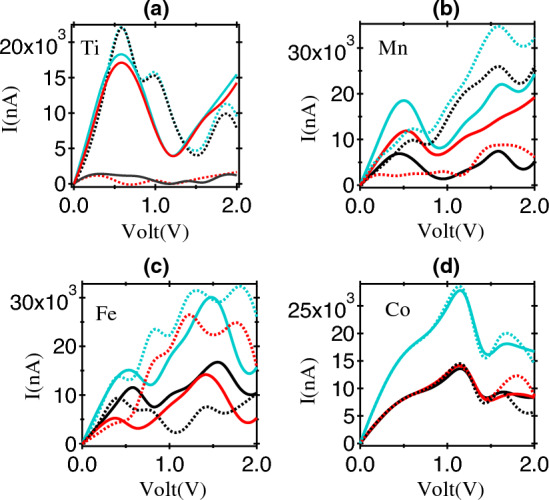
The spin-polarised I–V characteristics of (**a**) Ti, (**b**) Mn, (**c**) Fe and (**d**) Co based MTJ structures in the P and AP configuration. The solid lines represent the total current and dotted lines indicate individual spin contributions (blue = total, black = spin - ↑ and red = spin - ↓ contributions).

**Figure 5:**
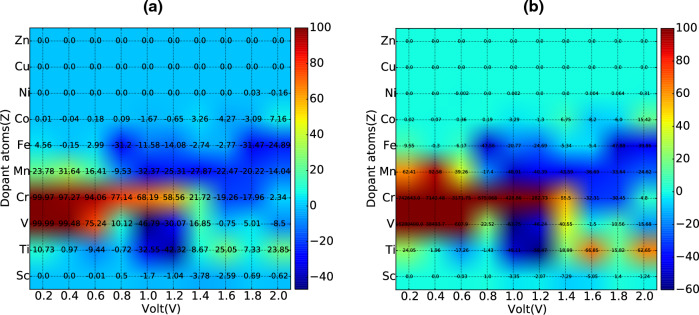
The 2D colour map representing the MR behaviour of various Transition Metal substituted Phosphorene MTJ structure as function of applied bias in the (**a**) pessimistic and (**b**) optimistic definition.

### MR at finite-bias

In the presence of a finite bias across the MTJ, the MR is estimated by following the recipe given in Eqs. (9–10). A sample case representing the conduction behaviour of V-substituted system in illustrated in Fig. 3. The total current in the P/AP configuration in Fig. 3a is contributed by the majority and minority spin components given by,

11 $$\begin{aligned} \mathrm {I=I_{\uparrow }+I_{\downarrow }} \end{aligned}$$

where $I_{\uparrow }$ and $I_{\downarrow }$ are the currents from the spin-$\uparrow$ and spin-$\downarrow$ channels respectively. In the P-case, current increases from V = 0 V towards higher applied bias and above 0.74 V, it decreases upto 1.02 V, above which steep rise is observed. For the AP case a very small amount of conduction is observed upto $\sim$ 0.5 V, above which current increases rapidly followed by a reduction above 1.08 V. Both in the P/AP cases, periodic modulation of the IV-pattern in the total current can occur due to quantum confinement in reduced dimension as also observed earlier^21^. With an increase in applied voltage, the reduction in current is a signature of negative differential conductance (NDC) behaviour. NDC has potential application in high frequency generation and modulation. With the help of the transmission spectra at different voltages as given in Fig. 3(d, e), the spin-resolved NDC behaviour can be explained. In the AP configuration, the value of transmission from both the spin-$\uparrow$ and spin-$\downarrow$ channels between − 0.5 V to 0.5 V is finite and large, compared to similar values of transmission of respective spin channels at 1.4 V. It signifies a reduction in current from 1 V to 1.4 V despite an increase in the bias window. The value of transmission increases significantly at 1.8 V, leading to an increase in current. This explains the NDC response, which can be similarly explained for the P case using the changes in transmission spectrum at different applied bias. The contribution of individual spin components towards the total current is given in Fig. 3b (P case) and Fig. 3c (AP case). In the P configuration, majority of the total current is contributed by the spin-$\uparrow$ channel while in the AP case, both the spin channels contribute significantly with spin-$\uparrow$ being the majority at higher applied bias. Periodic modulation in the IV-response is observed in individual spin currents with the presence of NDC in P/AP configuration. The significant difference between the spin-$\uparrow$ and spin-$\downarrow$ currents in Fig. 3b suggests the negligible presence of minority carriers in the total current. In such cases, a very large value of spin injection is observed offering a nearly perfect $\sim$100% spin polarisation, which can be very useful in perfect spin filtered devices. The electronic structure calculation reveals the existence of a half metallic phase in V-substituted system, which is responsible for the observed behaviour. The spin dependent conduction for Cr base MTJ system also offers similar interesting observations which is given in Fig. S1 in the SI^22^.

The spin-polarised conduction behaviour of Ti substituted MTJ system is shown in Fig. 4a. For the P case, spin-$\downarrow$ is the majority carrier while in the AP case it becomes minority mode of conduction. Such a switching of the majority spin carrier type with the change between P/AP case is a striking feature and observed in the case of phosphorene based system for the first time. It manifests in interesting NDC features and spin dependent conduction oscillation. The NDC in the P configuration is primary contributed by the spin-$\downarrow$ channel, while no major oscillations are observed in the spin-$\uparrow$ current. Similar scenario is realised in the AP case where NDC is solely contributed by the spin-$\uparrow$ components. Such a spin dependent NDC response can originate with the change of majority spin carrier type between P and AP cases^38^. The transmission spectrum in respective cases is given in Fig. S2^22^. The IV-behaviour for Mn, Fe, Co based MTJ system are given in Fig. 4(b – d). The conduction oscillation and NDC features are present in all these cases.

For the case of Mn, when measuring the current in parallel spin alignment, a monotonous increase is observed from 0 V to 0.5 V. Subsequently, the current exhibits a decreasing trend in the voltage range of 0.5 V to 0.9 V, characterized by a Peak-to-Valley Ratio (PVR) of 2.27 and a switching efficiency (S_E_) of 2.59 $\times$ 10^4^ nAV^-1^. The current then experiences a rise from 0.9 V to 1.6 V, followed by a decline in the voltage range of 1.6 V to 1.82 V. Current increases once again in the voltage range of 1.82 V to 2 V. Notably, the current is lower for anti-parallel spin alignment compared to parallel spin alignment at low bias (0 V to 0.76 V). However, beyond 0.76 V, the anti-parallel spin current surpasses the parallel spin current from 0.76 V to 2 V. The total current in anti-parallel spin configuration gets predominant contribution from spin-$\uparrow$ carriers, while in parallel spin alignment, the majority of the current arises from spin-$\downarrow$ carriers. For Fe, the parallel and anti-parallel spin currents are equal in voltage range of 0 V to 0.41 V. Subsequently, the parallel current surpasses the anti-parallel current in the voltage range of 0.41 V to 0.61 V. From 0.61 V to 1.46 V and from 1.55 V to 2 V, the anti-parallel current becomes higher than the parallel current. The anti-parallel current displays an oscillatory nature, while the parallel current exhibits NDC effects at bias voltages of 0.52 V (PVR = 1.24, S_E_ = 1.10 × 10^4^ nAV^-1^) and 1.48 V (PVR = 2.08, S_E_ = 3.71 × 10^4^ nAV^-1^). In the context of anti-parallel spin alignment, the majority current is spin-$\downarrow$ current, whereas in parallel spin alignment, the primary contribution originates from spin-$\uparrow$ carriers. In the case of Co, the parallel and anti-parallel currents are equal in applied voltage range of 0 V to 1.54 V. Beyond this, the anti-parallel current surpasses the parallel current in the voltage range of 1.54 V to 1.88 V, and from 1.88 V to 2 V, the parallel current becomes greater than the anti-parallel current. The current exhibits a continuous increase with rising voltage from 0 V to 1.14 V. Following this, there is a decrease in the voltage range of 1.14 V to 1.44 V, indicating NDC with a PVR of 1.83 and S_E_ = 4.34 $\times$ 10^4^ nAV^-1^. Subsequently, the current started to rise again in voltage range of 1.44 V to 1.68 V, and decrease in the voltage range of 1.68 V to 2 V. In the voltage range of 0 V to 1.54 V, the total current in both parallel and anti-parallel configurations predominantly arises from an equal contribution of spin-$\uparrow$ and spin-$\downarrow$ currents.

The 2D colour maps representing the MR values for different substitutional atoms as function of applied bias in different MTJ systems are shown in Fig. 5(a, b). In the optimistic definition, very large values of MR reaching upto $\sim 10^{7}$ is estimated for V. In the presence of Cr, maximum MR of 7.4$\times 10^{5}$ is observed at 0.2 V, which decreases with an increase in the applied bias and becomes negative at 1.6 V. Similar reduction in MR is also found for V based MTJ. With an increase in voltage, spin carriers gain additional energies which can contribute towards an enhanced scattering at higher applied bias, leading to a reduction of the MR value. The switching in the sign of MR is related to the change of relative current contributions from the P and AP configuration. In Fig. 5b, the negative MR values are primary centred at higher applied bias while the positive MR signal is found in the lower voltage region. Moderate values of MR were observed in the presence of Fe, Mn, and Ti in phosphorene, while almost negligible presence of MR is found in the cases of Zn, Cu, Ni, Co, Sc based MTJ. In the Pessimistic definition of MR in Fig. 5a, a similar behaviour of the MR is obtained with the maximum MR values $\sim$100% observed for V and Cr based MTJ at low applied bias. The decrease in MR value with voltage and change in sign is also present here. The contrast of MR in different regions of the phase diagram is slightly smaller in the pessimistic case compared to Fig. 5a. The very large value of MR in the V and Cr based MTJ systems suggests their usefulness for various spintronic applications such as magnetic random access memory (MRAM), spin switches and sensitive MR sensors. The uniqueness of the current investigation is that it offers tunable MR response over a large range of substitutional atoms and applied bias which can be chosen as per desired applications. Common MTJ structures suffer from conductance mismatch at the FM/tunnel barrier interfaces which can be mitigated in an all phosphorene based MTJ structure as explained in the current work. Apart from the uniqueness in design, such MTJ structure offers colossal MR response comparable to that of reported values. It offers a new perspective in nano scale MTJ design and paradigm shift in 2D planar spin valves.

**Figure 6:**
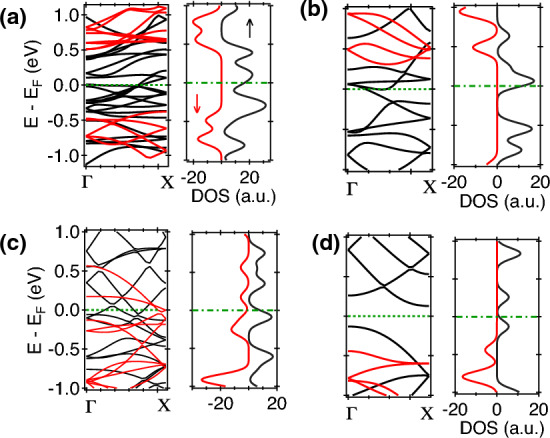
The band-structure and DOS of V based MTJ structure : (**a**) central scattering region and (**b**) electrode. Same for Cr based MTJ device of the (**c**) central part and (**d**) electrode.

The origin of high MR in V and Cr based MTJ can be traced within their electronic behaviour as shown in Fig. 6. The band structure and density of states for the electrode and the central scattering region for V based MTJ is shown in Fig. 6(a, b). It was observed that the electrodes and the scattering region are half metallic in nature with similar spin type being the majority carrier^27^. A similar observation is also made for Cr-MTJ^24^. The absence of the minority spin channel in the conduction offers drastic difference of resistances between the P and AP configuration, leading to a high MR for V-MTJ. For Ti MTJ, the electrodes are half metallic (HM) with spin-$\uparrow$ as the majority carrier while the central part is metallic with more available states for the spin-$\downarrow$ component. In such scenario, the spin-polarised carriers from the electrodes will face a larger scattering in the conduction process. Moreover the spin density of state (SDOS) value at the Fermi level of the electrodes are comparably small, having fewer carriers for spin injection. A combination of these two is responsible for small MR values for Ti-MTJ. Interestingly, the predominant presence of negative MR values can be related to the presence of spin-$\downarrow$ states being the majority carriers. For Zn, Cu, and Ni based MTJ, the central part is non-magnetic in nature while the electrodes offer very small intrinsic spin-polarisation. For Co and Sc-MTJ, the electrodes are semiconducting in nature which offers low conductivity at the source. All these lead to a very low MR signal for such substitutional configuration spin valves. A phase table detailing the nature of electronic states of the electrode and central scattering part for individual substituted atoms is given in Table S1 in the SI^22^. Existing studies with graphene, silicene, and phosphorene based MTJ structures are made by sandwiching the 2D layer between two ferromagnets that led to the observation of charge conductance fluctuation, spin-transfer torque etc.^23,28,36^. The theoretical studies in such MTJ systems offered a MR ratio: (i) $\sim$ 107% for Ni/phosphorene^34^, (ii) $\sim$ 100% for CrO_2_/graphene^26^, (iii) $\sim$ 3200% for CrO_2_/graphene-boron nitride^31^ and (iv) $\sim$ 850% for MoS_2_/VSe_2_^29^ etc. In comparison to this, the MR values reported in the present work are significantly higher or comparable in respective cases.

**Figure 7:**
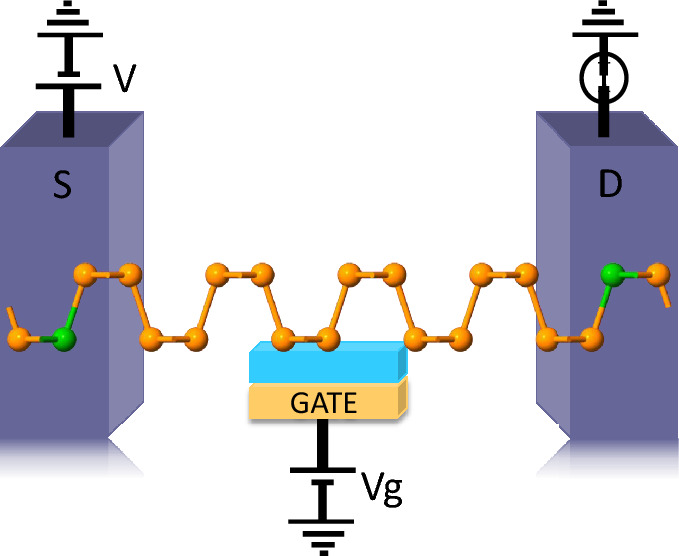
Schematic representation of MTJ structure in the presence of a gate Voltage.

### Effect of gate voltage

**Figure 8:**
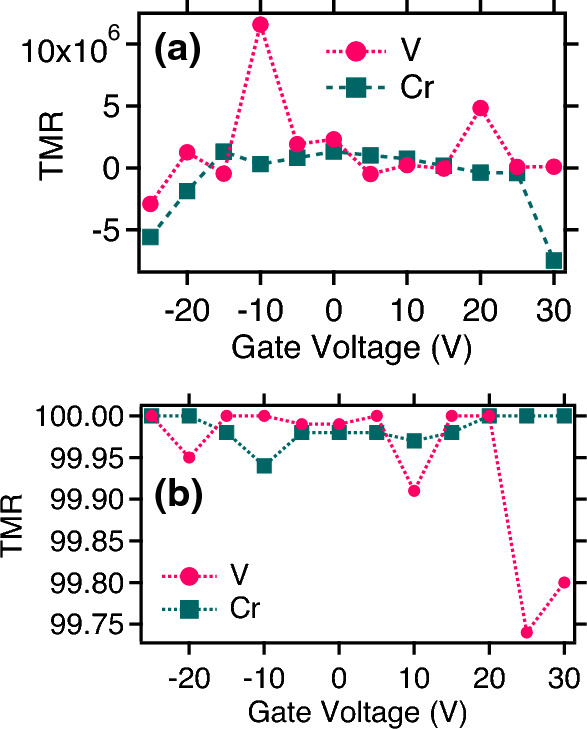
The MR ratio at different gate voltages in the zero applied bias case for V and Cr-based MTJ in the (**a**) Optimistic and (**b**) Pessimistic definition.

In order to understand the role of a gate voltage on the observed MR response, we have constructed an MTJ structure with our all phosphorene layer as the base element in a field effect transistor (FET) geometry as shown in Fig. 7. The gate voltage ($V_g$) offers local control over the carrier density in the channel area, which can also influence the spin dependent conduction through it. In the zero bias case, the MR reduces on both sides of $V_g$ = 0 V to a higher gate voltage. For V based MTJ, at very high gate voltage the MR becomes negative which for example at $V_g$ = 30 V is − 7.489 $\times 10^{6}$ and at $V_g$ = − 25 V takes a value of − 5.6$\times 10^{6}$, which is shown in Fig. 8(a, b). Similar trend is also observed for the case of Cr based MTJ. However some spurious MR peaks are observed at $V_g$ = − 10 V and 20 V with MR values $\sim$ 1.157 $\times 10^{7}$ and 4.83 $\times 10^{6}$ respectively.

**Figure 9:**
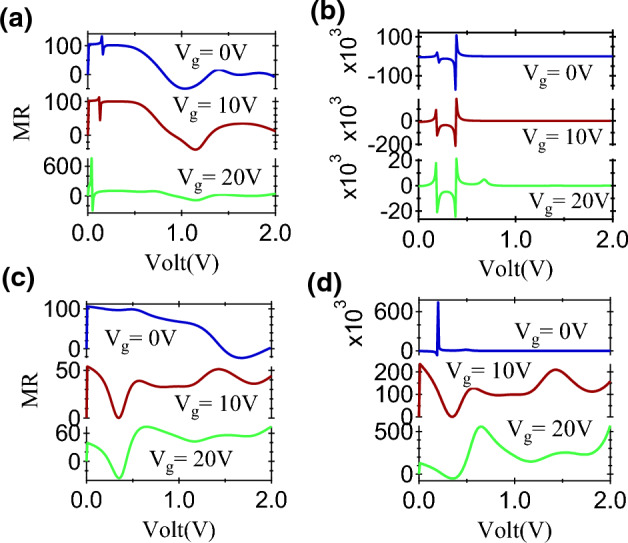
The (**a**) Pessimistic and (**b**) Optimistic MR ratio for Vanadium based MTJ and (**c**) Pessimistic and (**d**) Optimistic MR ratio for Cr based MTJ at different values of $V_g$.

The spin-polarised I–V curve of Cr and V based MTJ in the P and AP configuration is shown in Fig. S4 in the SI^22^. For Cr, the total current in the AP configuration is reduced at higher voltages while it did not show a drastic change for V case. However the total current in the P configuration for both these cases improved significantly in the presence of the non-zero gate voltage. In the P configuration, a colossal enhancement in conduction ( $\sim$ 160% at $V_g$ = 10 V ) is observed for Cr based MTJ from the $V_g$ = 0 V configuration, which corresponds to a change in current $\sim 27.6\ \mu A$. This change is also significantly visible for V based MTJ with conduction enhancement of 32.63% ($V_g$ = 10 V) and 25.4% ($V_g$ = 20 V) respectively. The relative contribution of individual spin channel stays similar with the change in gate voltage.

It was observed that the size of $\mathrm {MR_{O}}$ is reduced in the presence of the positive gate voltage for Cr-MTJ. At non-zero $V_g$, modulation of MR is observed upto 2.0 V, which is shown in Fig. 9. At $V_g$ = 10 V, the maximum MR is $\sim 235$% while at $V_g$ = 20 V, a maximum MR $\sim$ 560% is estimated. For V based MTJ, the MR values stay significantly high in the presence of a non-zero gate voltage. A similar MR pattern is observed at low applied bias with MR changing its sign between positive and negative values. In the pessimistic definition, a positive to negative switching in MR is observed for V-MTJ independent of the gate voltage. The highest MR value $\sim 760\%$ is observed at $V_g$ = 20 V while the maximum stays around $\sim 132\%$ at $V_g$ = 10 V. The MR signal in the pessimistic definition for Cr-MTJ shows similar features at $V_g \ne$ 0 V. The maximum $\mathrm {MR_{P}}$ are $\sim$106%, 54% and 74% at $V_g$ = 0 V, 10 V and 20 V respectively. These observations indicate that MR response can be tuned in the presence of a gate voltage. It offers an additional degree of freedom which can be very beneficial for the development of all 2D spin architecture and future spintronic integration towards quantum technology.

## Conclusion

In this work, we have designed and investigated the behaviour of an all phosphorene based 2D-spin valve structure with electrodes made through substitution of a range of 3d block elements in the left and right regions of phosphorene nanoribbon (mPh-Ph-mPh). First-principles based investigation revealed the spin-dependent characteristics of electronic transport in these structures which can be related to the electronic structure of the substituted system. It includes observation of a spin-dependent negative differential conductance (SDNDC) behaviour, conductance oscillations and switching of the majority spin character between the P and AP configurations of the magnetic regions of mPh-Ph-mPh structure. In the case of Ti and V-substituted MTJ systems, a very large value of MR $\sim 10^6$ was estimated and it is significant for Mn, Ti, Co and Fe-substituted systems as well. Detailed exploration of the dependence of MR with applied bias and type of substitutional element was performed through a 2D phase diagram in the pessimistic and optimistic definition, which offers an opportunity to engineer the MR response. Further control of the MR signal is achieved by applying a local gate voltage in the channel area, which also allows a positive to negative crossover with a change in V_g_. The unique design of the MR structure offers design advantage of an all phosphorene single layer MR structure. The tuning and control of the MR through different substituted elements presents engineering capabilities of such MR structures for various spintronic applications.

### Supplementary Information

Supplementary Information.

## Data Availability

The datasets used and/or analysed during the current study available from the corresponding author on reasonable request.
