# Supplementary material for: An all phosphorene lattice nanometric spin valve
Source: Sci Rep. 2024 Apr 21;14:9138. doi: 10.1038/s41598-024-58589-4 (PMC11033266; doi:10.1038/s41598-024-58589-4)
Supplement: Supplementary file 1 — Supplementary Information. [file 41598_2024_58589_MOESM1_ESM.pdf]

Supporting Information

An all Phosphorene Lattice Nanometric Spin Valve

P. Kumari[1], S. Majumder[1], S. Kar[1], S. Rani[1], A. K. Nair[1], K. Kumari[1], M. Venkata Kamalakar[2], S. J. Ray[1]

[1]Department of Physics, Indian Institute of Technology Patna, Bihta 801103, India

[2]Department of Physics and Astronomy, Uppsala University, Box 516, SE-75120, Uppsala, Sweden

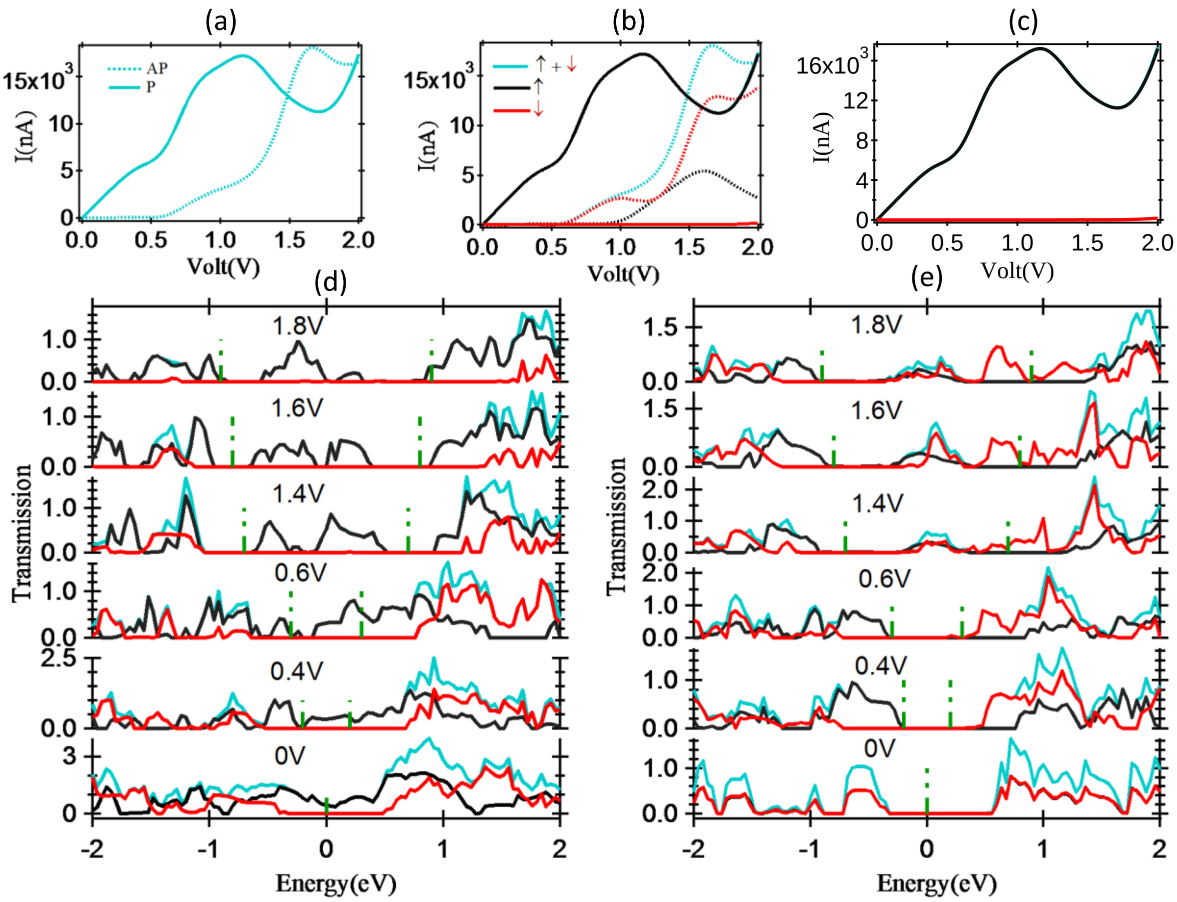

Figure S1: The I-V relationship for Cr based Phosphorene MTJ device in the (a) P and AP configuration, (b-c) various spin-splitted components, the solid (dotted) line represents the total current and P(AP) configuration, (d)Transmission spectrum at different bias voltages in P and (e) AP configuration.

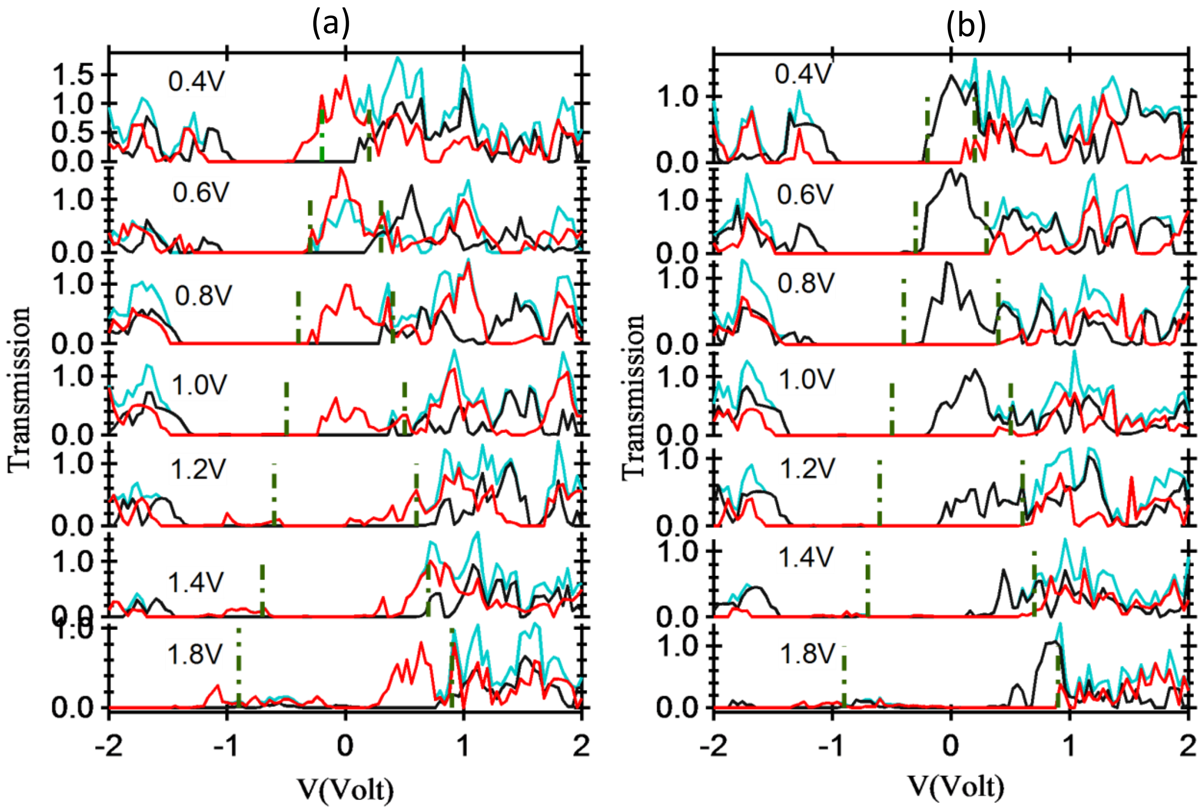

Figure S2: The transmission spectra of Ti-based MTJ device at different applied bias in the (a) P and (b) AP-configuration.

Table S1: Phase table demonstrating various electronic phases of the electrodes and scattering regions of various MTJ structures

Doping Element	Nature of electronic state	
	Electrode	Scattering region
Sc	Semiconductor	Non-magnetic metal
Ti	Half Metal	Magnetic Metal
V	Half Metal	Half Metal
Cr	Half Metal	Magnetic Metal
Mn	Magnetic Metal	Magnetic Metal
Fe	Magnetic Metal	Magnetic Metal
Co	Semiconductor	Magnetic Metal
Ni	Magnetic Metal	Non-magnetic metal
Cu	Magnetic Metal	Non-magnetic metal

Zn	Magnetic Metal	Non-magnetic metal

S1. Negative Differential Conductance (NDC)

A sample I-V curve representing the NDC response is given in the figure below (Fig. S3), where the solid line represents current in parallel configuration and the dotted line represents the current in anti-parallel configuration. The current-voltage behaviour in both the configurations is oscillatory. For the purpose of the device application, we calculated two physical quantities from the spin-current. First one is peak to valley current ratio (PVR), which is expressed by,

$$\mathrm{PVR} = \left| \frac{\mathrm{I_{peak}}}{\mathrm{I_{valley}}} \right|$$

and the second one is switching efficiency (S_E), which is defined as,

$$\mathrm{S_E} = \left| \frac{\mathrm{I_{peak}} - \mathrm{I_{valley}}}{\mathrm{V_{peak}} - \mathrm{V_{valley}}} \right|$$

Where I_{peak} , V_{peak} and I_{valley} , V_{valley} are the currents and voltages in the peak and valley positions of the I-V curves, where point (a), (d) are the valley positions and (c), (b) are the peak positions. The value of PVR for P configuration is 3.71 and 1.78 in AP configuration. Similarly, the value of S_E for P configuration is 18.344 mS and 22.1 mS for AP configuration. PVR simply explains the ON/OFF ratio of the current between the peak and valley position and S_E explains how fast switching occurs between the peak (ON) and valley (OFF) regions.

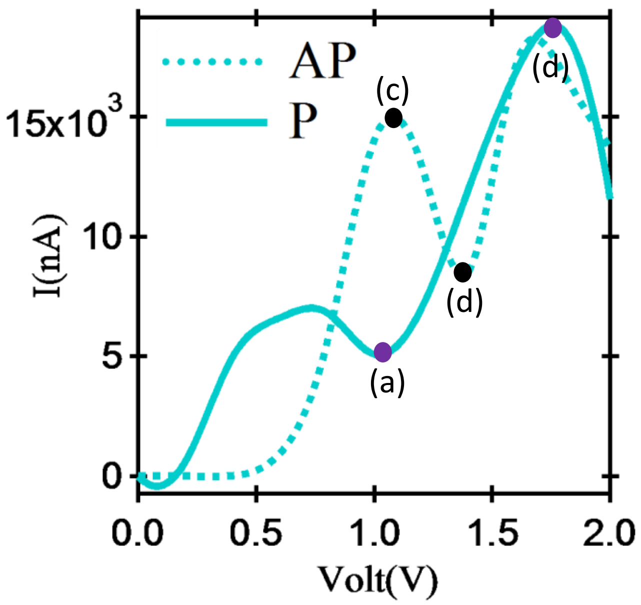

Figure S3: The I-V curve of the V based MTJ in the P and AP configuration.

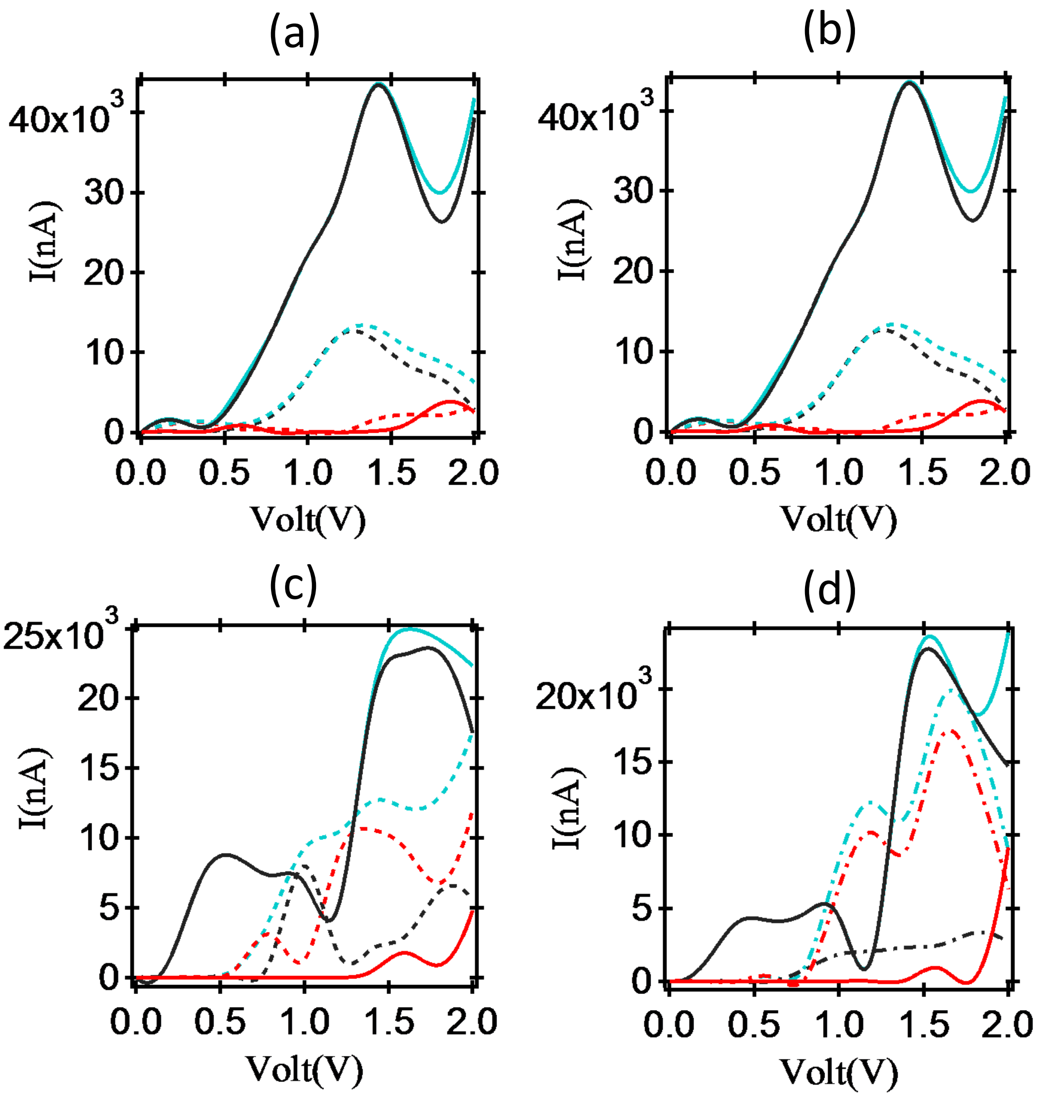

Figure S4: The I-V curve of Cr-based MTJ at V_g = (a) 10V, (b) 20V, (c) -10V, (d) -20V. Blue, black and red colour signify the total, spin-up and spin-down currents respectively, while the solid (dotted) line represents total current in P(AP) configuration.

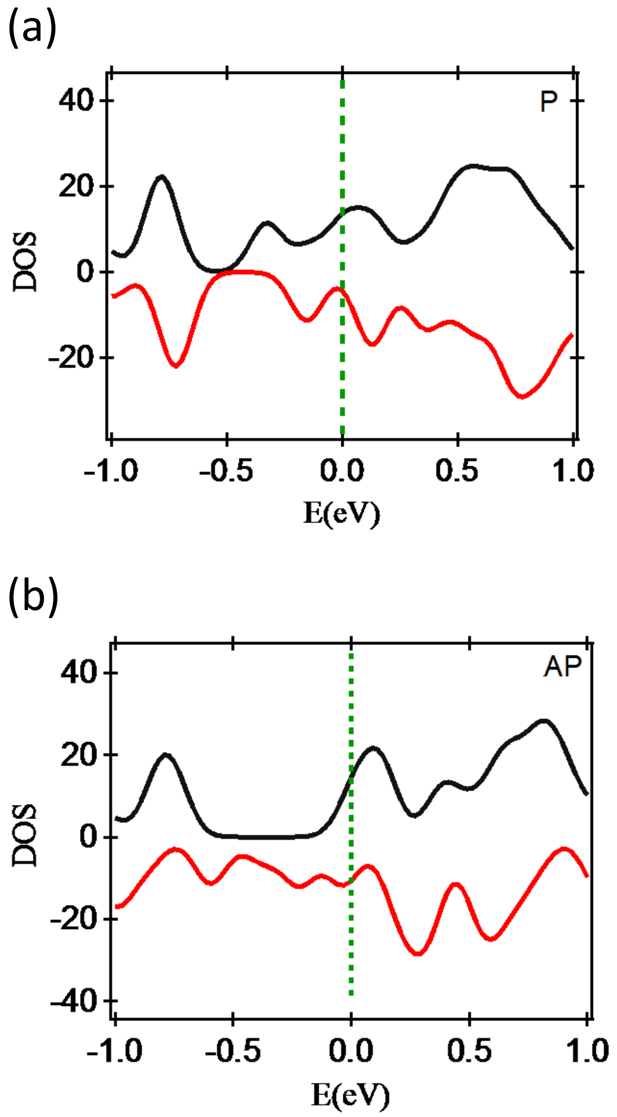

Figure S5: The DOS graph of scattering region for Ti based MTJ in (a) P configuration and (b) AP configuration. Red and black lines represent the contribution from up and down spin states respectively.

S2. Structure Stability and additional doping Configuration

The stability of the substituted phosphorene system was studied by investigating the formation energy defined by,

$$E_{form} = (aE_{TM} + bE_{phos} - E_{TM+phos})/(a + b)$$

Where a and b represent the number of substituted atoms and phosphorous atoms, E_{TM}, E_{phos} are the total energies of transition metal atom and phosphorous atoms respectively and $E_{TM+phos}$is the total energy of the substituted phosphorene layer. This equation has been used for estimating formation energy for

various 2D systems **[RSC advances 6, no. 13 (2016): 10919-10929, Materials & Design 121 (2017): 77-84, Physics Letters A 380, no. 40 (2016): 3270-3277]**.

Here, we have created different possible configurations (configuration – 1, 2, 3 as shown in Fig. S6) and estimated the formation energy. Following the referee's suggestion, we have followed the method mention in **Physical Review B 75, no. 15 (2007): 153401** and estimated the formation energy of similar substituting configurations in graphene. The comparison is shown in Fig. S6(g). It can be observed that in similar substituting configuration, the transition metal substituted phosphorene has lower formation energy compared to the graphene doped system for various dopant atoms in most cases. It indicates that substitution of 3d – block elements in phosphorene is more energetically favorable than in graphene. This suggests the stability of the present substituted systems. As we are not dealing with a carbon-based material, hence no hydrocarbons can be formed for the present system.

Moreover, the formation energy varies between the range of 3.5 – 4.3 eV/atom (Configuration-1, 2, 3). The maximum is observed for Ni substituted phosphorene (4.28 eV/atom) and minimum for Zn-doped Phosphorene (3.53 eV/atom). These values are comparable to that of similar 2D materials like; graphene (7.85eV/atom), Fe_2Si (4.10 eV/atom) (**Nano letters, 17(5), 2771-2777),** Ni_2X (X=Si/Ge) (4.80 eV/atom and 4.42 eV/atom respectively for X=Si and Ge) **(Physical Chemistry Chemical Physics, 17(39), 26043-26048),** MnB (4.80 eV/atom) **(Nanoscale Horizons, 3(3), 335-341)** etc. The above observation suggests that substituting configurations considered in this work are stable in the presence of different dopant atoms.

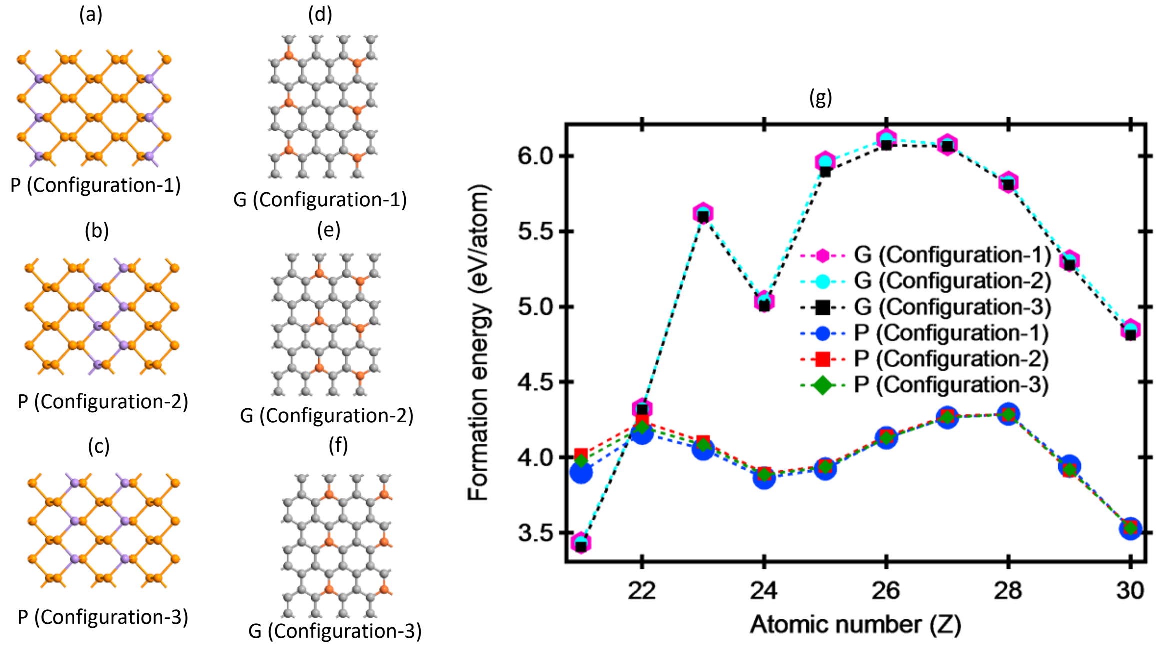

Figure S6: The top view of a (a-c) phosphorene and (d-f) graphene layer with dopant atoms in various substituting configurations. (g) The formation energy as a function of atomic number (Z) of the substitutional atom, where G represents the graphene and P stands for phosphorene.

S3. Phonon band structure

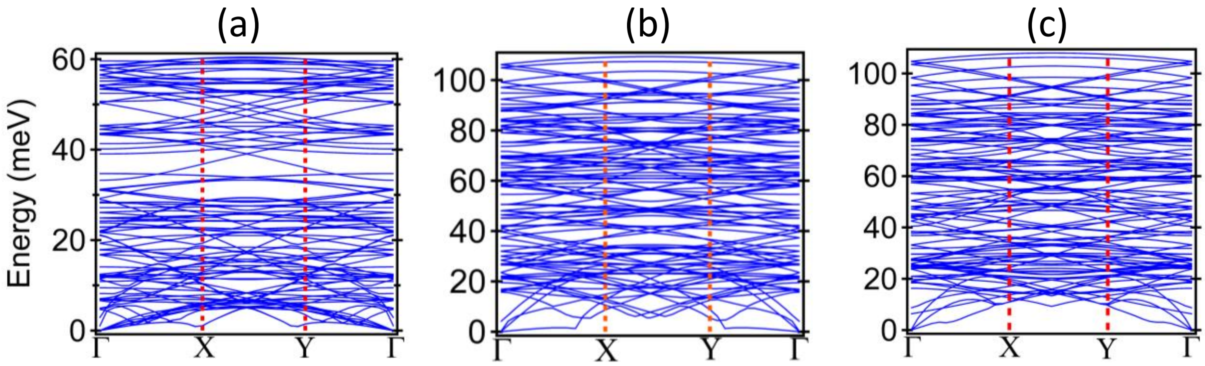

Figure S7: The phonon band structure (a) V substituted configuration, (b) Cr substituted configuration, and (c) Mn substituted configuration.

S4. Estimation of critical temperature (T_c) for left/right electrode

We have calculated the T_c of right/left electrode through mean field approximation of the Heisenberg model, which is defined as **[Physical Chemistry Chemical Physics 21, no. 42 (2019): 23713-23719]**,

$$T_c = -\frac{2}{3}\frac{\Delta E}{NK_B}$$

Where N is the number of substitutional impurities for each 3d – block metal atom and k_B is the Boltzmann constant. ΔE is the energy difference in between ferromagnetic (FM) and anti-ferromagnetic (AFM) configuration.

Table S2: The T_c for various 3d - block element doped electrodes

Doping Element	T_c (K)
Ti	465.42
V	557.99
Cr	1482.54
Mn	329.75
Fe	464.39

The estimated T_c value for various cases are shown in Table S2. The T_c for Ti, V, Cr, Mn and Fe systems are higher than the room temperature and the Sc, Cu and Zn doped system is non-magnetic is nature, while for Co and Ni cases the energy value is approx. equal in FM and AFM states.

S5. Relative stability in the presence of various transition metals and configurations designed with same set of atoms

The formation energy was estimated for our proposed structure and compared with other compounds or configurations that can be made with the same set of atoms. The same set of atoms have been considered

in the black phosphorene with 5 allotrope of phosphorene, that is blue phosphorene, red phosphorene, red phosphorene, γ – phosphorene, δ – phosphorene and θ_0 – phosphorene. These phosphorene allotropes are used as the building blocks to make the initial configuration with 9 additional configurations as shown in Table S3.

In the present work, the calculated binding energy is difference between the total energy of compound and a pseudoatomic calculation done with same cut off, which is mentioned in graphane paper [**Physical Review B, 75(15), 153401**]. Our cut off energy is 180 Ry. The pseudoatomic total energies are -260.23eV, -379.68eV, -259.64eV and -613.89eV for phosphorous (P), vanadium (V), chromium (Cr) and manganese (Mn). The binding energy is as low as for black phosphorene with transition metal atom (V, Cr, Mn) as compared to various configuration in other allotrope of phosphorene [see Figure S8]. This is a good sign to use black phosphorene of our proposed TMR structure from the other allotropes of phosphorene. The 7th configuration is more favourable out of these 10 configurations of black phosphorene with V, Cr and Mn atoms. The lowest binding energies are estimated at -4.10eV/atom, -3.89eV/atom, -3.94eV/atom for V, Cr and Mn cases. The binding energies for our transition metal based black phosphorus TMR structure are only 0.04eV/atom, 0.02eV/atom and 0.02eV/atom higher than the lowest binding energy in black phosphorene case with V, Cr and Mn atoms respectively. Our structural stability is also mentioned in our previous manuscript [**Physical Chemistry Chemical Physics, 22(10), 5893-5901, Physical Chemistry Chemical Physics, 21(42), 23713-23719**]. The stability of suggested TMR structure from the other compounds is explained by the formation energy analysis. The primary reference of our TMR structure is bulk phosphorous. We have used same computational parameters and methodology to calculate the binding energy of -3.76eV/atom for bulk phosphorous. The formation energies per atom for various transition metal (V, Cr, Mn) compound with phosphorous atom are shown in Figure S9, as a function of atomic percentage of transition metal atom. The point at (0, 0) corresponds to phosphorous in this plot. The negative value of formation energy explains the high stability of our proposed TMR - structure. Our, Cr – based TMR structure is more stable than the other compounds of chromium with phosphorous atom, as shown in Figure S9(b). We have made the various substitution configurations of our proposed phosphorene planar spin valve structure, where the electrodes are heavily TM atom substituted, but the positions of substitution of TM atoms have changed. The estimated MR values in various substitution configurations for vanadium atoms in the zero - bias case are listed in Table S4. In every substitution configuration, MR is estimated to be relatively high, close to the perfect spin-filtering performance. We have found that the nature of MR remains unchanged as we vary the substitution positions of the TM atoms within the electrode region. In Table S5, we have slightly increased the substitution concentration of TM atoms and estimated MR value at zero-bias by varying the substitution position of the vanadium atom in electrodes. The nature of MR value remains unchanged. The results show that even with a slight increase in substitution concentration of vanadium atoms in the electrode region, the MR properties remain the same, suggesting the robustness of the observed MR effect in the proposed structures independent of the position and concentration of the substitutional atoms.

Table S3: Various configuration with same set of atoms in different phosphorene allotrope with transition metal atoms.

Configuration	$\theta_0 - P$	Red - P	$\delta - P$	Blue - P	$\gamma - P$	Black - P
1	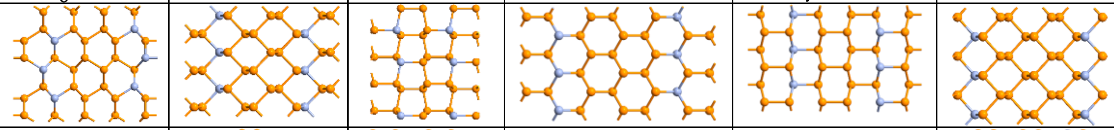					
Side view	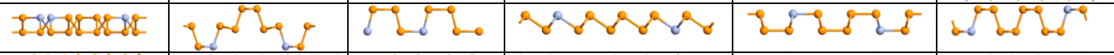					
2	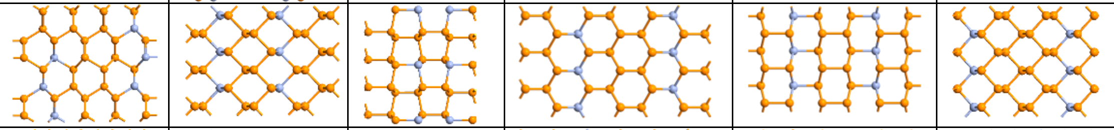					
3	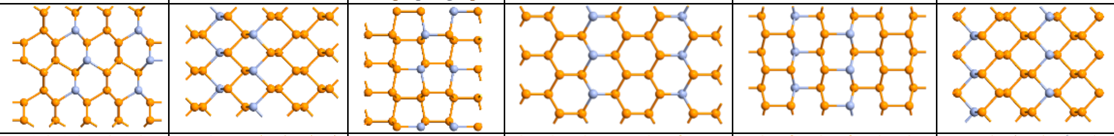					
4	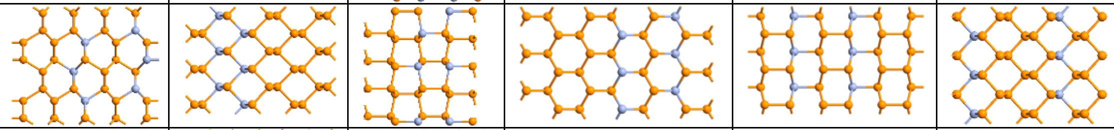					
5	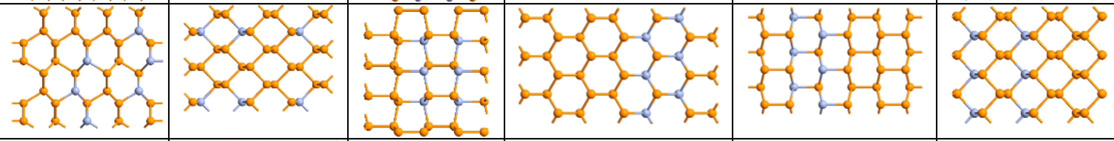					
6	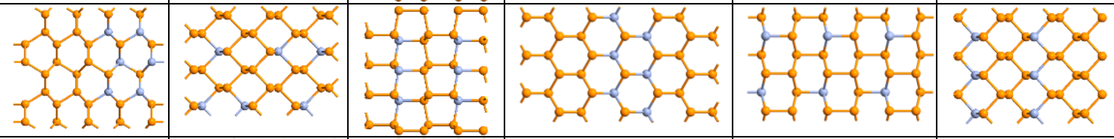					
7	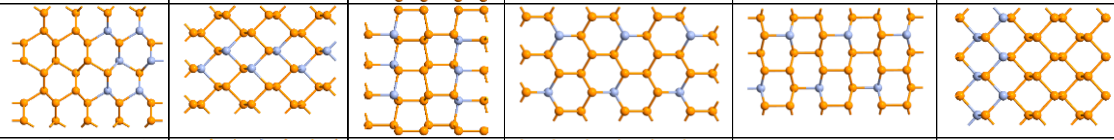					
8	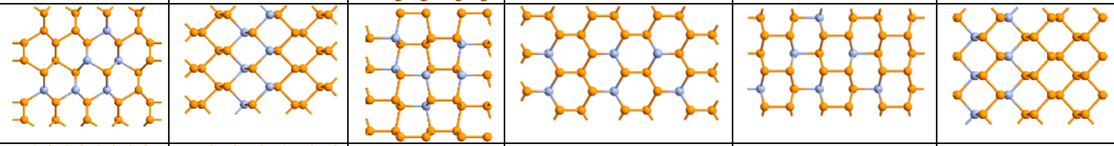					
9	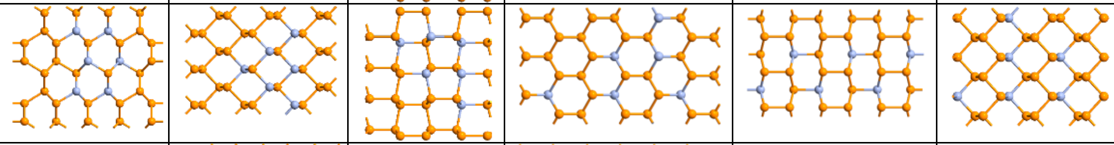					
10	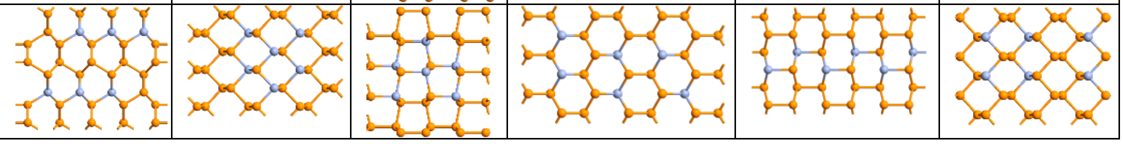					

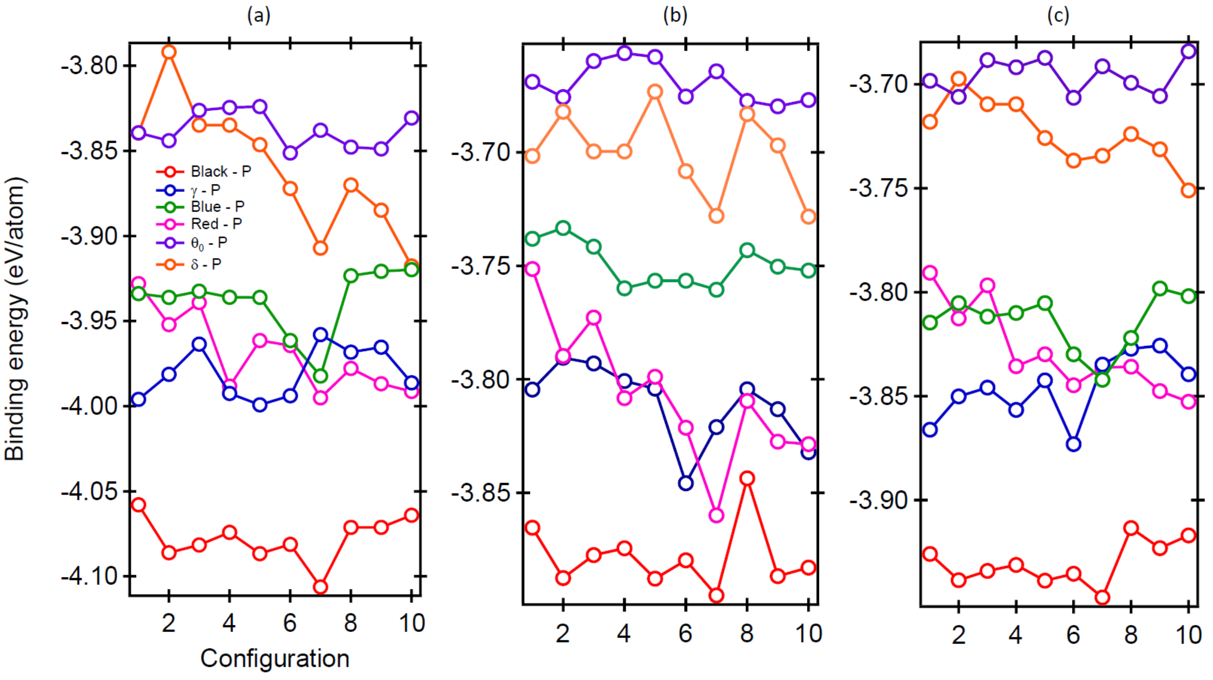

Figure S8: The binding energy of transition metal substituted phosphorene with same set of atoms. (a) Vanadium substituted, (b) Chromium substituted, (c) Manganese substituted.

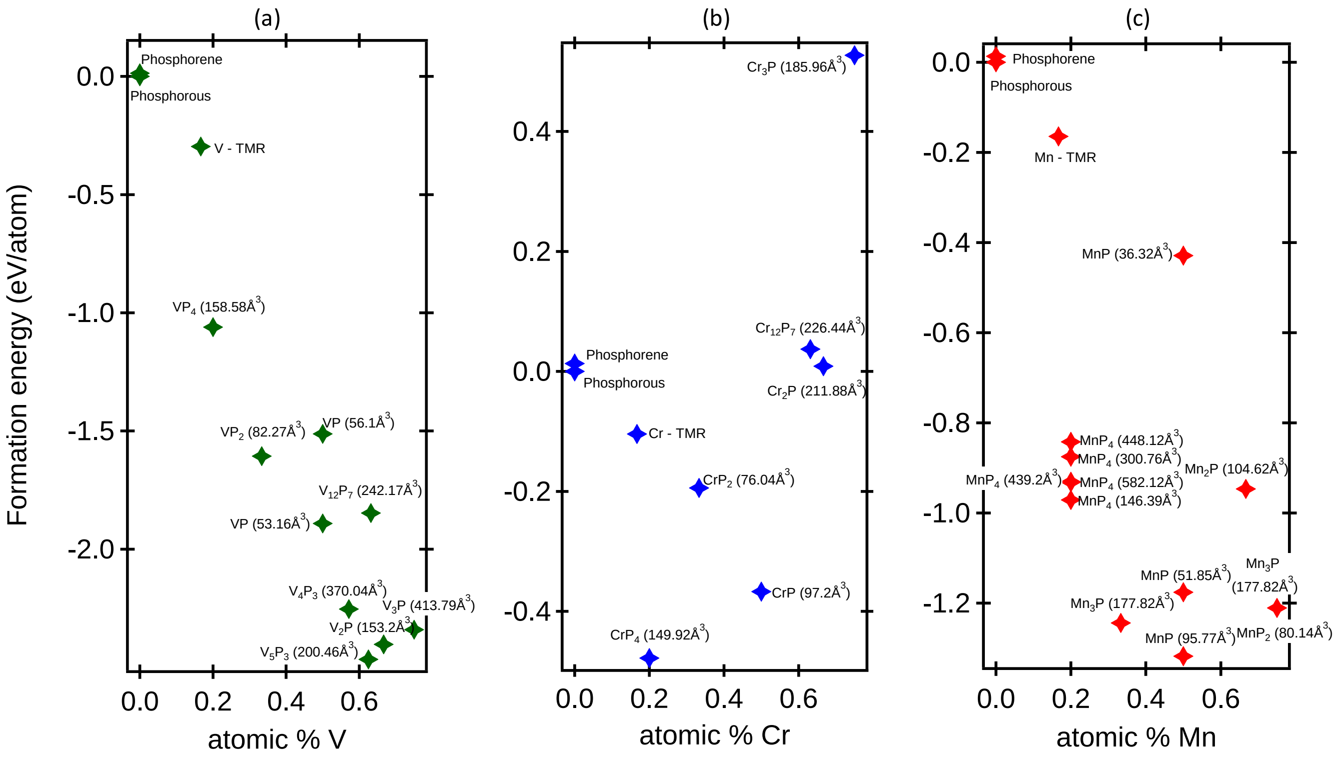

Figure S9: Formation energy as a function of atomic percent of transition metal atom. (a) Vanadium, (b) Chromium, (c) Manganese. The number in bracket represents the cell volume of compound.

S6. Estimation of TMR in variation of substitutional position of TM atom in electrode region:

Table S4: Various substitution position of V - atoms substituted in electrode region.

Configuration	Device Figure	Optimistic TMR (%)	Pessimistic TMR (%)
1	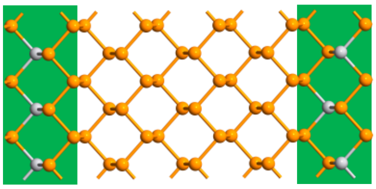	1348573.05	100
2	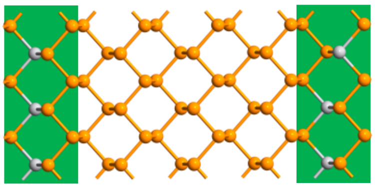	30816863.40	100
3	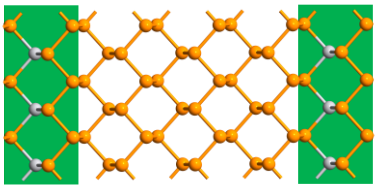	7168024.96	100
4	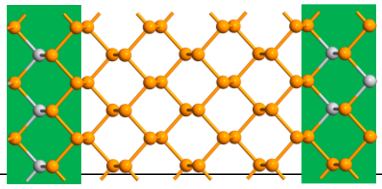	546614.19	100
5	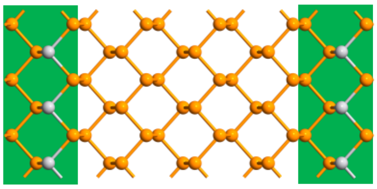	1611937.621	100
6	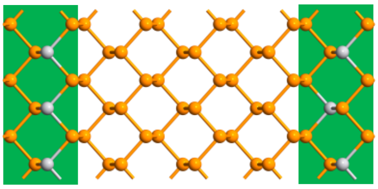	820062409.79	100
7	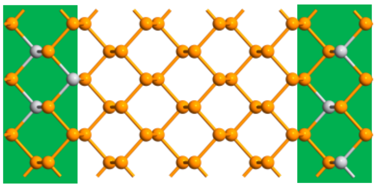	622056.47	100
8	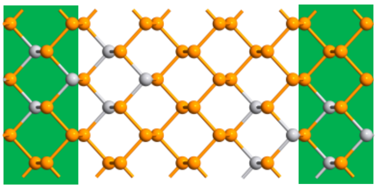	8085816.21	100

9	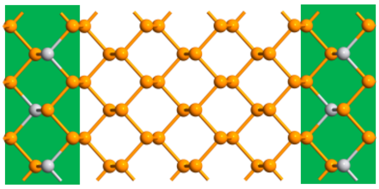	8667123.06	100
10	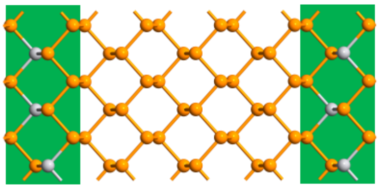	37995432.72	100

S7. Study the TMR with slightly increased substitution concentration of TM atoms in electrode region:

Table S5: various substitution configuration of slightly increased substitution TM atoms in electrode region.

Configuration	Device Structure	Optimistic (TMR)	Pessimistic (TMR)
1	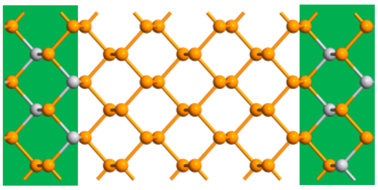	1550.45	88.57
2	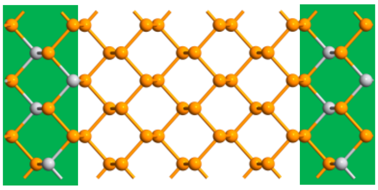	318336.88	99.94
3	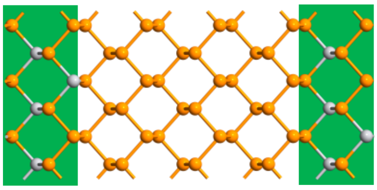	5505397.20	100
4	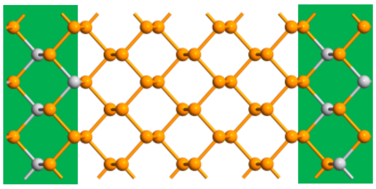	72574.74	99.73
